# Localized histopathological effects of high-salt intake on the aorta and kidney in rats

**DOI:** 10.3389/fphys.2025.1659918

**Published:** 2025-09-24

**Authors:** ChangHwa Oh, Sung Jun Hong, Jeonghun Kim, Yu-Gyeong Kim, Hye-Won Lee, Dokyoon Kim, Eunkyoung Park, Taekyung Kim, Young-Min Shon

**Affiliations:** ^1^ Biomedical Engineering Research Center, Samsung Medical Center, Seoul, Republic of Korea; ^2^ Department of Medical Device Management and Research, SAIHST, Sungkyunkwan University, Seoul, Republic of Korea; ^3^ AI Research Center, Samsung Medical Center, Seoul, Republic of Korea; ^4^ Department of Digital Health, SAIHST, Sungkyunkwan University, Seoul, Republic of Korea; ^5^ Department of Biomedical Engineering, Soonchunhyang University, Asan, Republic of Korea; ^6^ Department of Neurology, Samsung Medical Center, Sungkyunkwan University School of Medicine, Seoul, Republic of Korea

**Keywords:** high-salt diet, heart, aorta, kidneys, histopathology

## Abstract

**Background:**

Excessive dietary sodium intake is a major global public health concern, responsible for approximately 1.89 million deaths annually. High salt consumption disrupts systemic homeostasis and elevates blood pressure, thereby increasing the risk of cardiovascular diseases, including arterial stiffness, atherosclerosis, heart failure, myocardial infarction, and stroke. Additionally, excessive salt adversely affects renal structure and function by inducing glomerular and tubular injury, promoting extracellular matrix (ECM) accumulation and fibrosis, ultimately accelerating chronic kidney disease.

**Objective:**

To investigate the histopathological changes and differences in the heart, aorta and kidneys of Sprague Dawley rats subjected to normal and high-salt diets.

**Methods:**

This experiment examined male Sprague Dawley rats, aged 6 weeks, which were divided into a normal diet group (control, n = 9) and a high-salt diet group (high salt, n = 10), and were observed over 12 weeks. Blood pressure (BP) changes were measured, and histological analyses of the heart, aorta and kidney tissues were performed.

**Results:**

Our results revealed localized histological alterations in the aorta and kidneys following high-salt intake. Aortic wall thickening was observed specifically at Zone 0 (A2, p < 0.05), without significant collagen deposition. In the kidneys, the high-salt group showed significant capsular space narrowing (p < 0.05), glomerular hypertrophy, and tubular dilatation, accompanied by increased interstitial collagen deposition (p < 0.0001), indicative of renal fibrosis. In contrast, no significant pathological or fibrotic changes were detected in the left ventricle aside from body weight–related variation.

**Conclusion:**

These findings indicate that a high-salt diet induces localized histopathological changes in specific regions of the aorta and kidneys. This study provides foundational data for understanding the pathological mechanisms of high-salt intake and offers insight into potential prevention strategies for salt-induced aorta and renal injury.

## Introduction

Excessive salt intake is associated with approximately 1.89 million deaths worldwide annually (Murray, 2020) and is widely recognized as one of the major health concerns globally. The global average salt intake is estimated to be between 9 and 12 g per day (He and MacGregor, 2010), which far exceeds the World Health Organization (WHO)’s recommendation of 5 g per day (Organization, 2023).

High salt consumption disrupts the sodium balance in the body, increasing the blood pressure (BP), a key contributor to both cardiovascular and kidney diseases (Muela, 2020).

Sodium draws water into the bloodstream, increasing blood volume and pressure on blood vessels, resulting in hypertension, a primary factor elevating the risk of cardiovascular diseases. (Baldo et al., 2015).

Specifically, excessive salt intake induces arterial wall thickening and collagen accumulation in the major arteries, such as the aorta, leading to atherosclerosis and arterial stiffness (Sawada et al., 2020).

This condition impairs blood vessels’ ability to expand and contract, further aggravating high BP and increasing strain on the heart (Berger et al., 2015). Over time, arterial stiffness is closely associated with hypertension and can result in serious complications, such as heart failure, myocardial infarction, and stroke (Baldo et al., 2015).

The ascending aorta dilation occurs as hypertension applies sustained stress to the aortic wall, causing structural remodeling and eventually expansion (Humphrey, 2021).

Conversely, the descending aorta primarily exhibits increased stiffness and vascular resistance under hypertensive conditions, resulting in the loss of aortic elasticity and higher vascular resistance, thereby worsening hypertension in a vicious cycle. This process affects the remodeling of vascular smooth muscle cells and vascular endothelial cells, mediated by changes in sodium and calcium transporters and elevated levels of reactive oxygen and nitrogen species. Consequently, the glycocalyx is damaged, increasing the sodium permeability within the cells and contributing to vascular dysfunction. The ascending aorta was primarily dilated, whereas the descending aorta was stiff with increased vascular resistance (Guettler, 2023).

Excessive salt intake also significantly impacts renal function. A highde-salt diet directly damages the glomeruli and tubules, resulting in impaired renal function and fibrosis (Teixeira et al., 2020). High-salt consumption promotes tubular atrophy and dilation, even without increased BP, resulting in a long-term increase in the risk of renal function loss (Gu et al., 2008), (Hayakawa et al., 2020). This process results in fibrotic tissue formation in the kidney, a major cause of chronic kidney disease (CKD), which impairs filtration capacity, leading to proteinuria and accelerated renal damage (Berger et al., 2015). High-salt intake further induces an inflammatory response that promotes mesangial cell proliferation within the glomeruli, resulting in structural changes and damage to the filtration barrier (Hayakawa et al., 2020; López-Novoa, 2011). These alterations affect the substance quantity and quality filtered into Bowman’s space (capsular space), ultimately impairing renal function. Excessive salt intake also increases extracellular matrix component accumulation, such as collagen, around the capsular space, forming the fibrotic tissue that narrows the filtration space, reduces filtration efficiency, and leads to proteinuria. Consequently, declined kidney filtration capacity accelerates damage (Khadive et al., 2021). Moreover, the effects of a high-salt diet extend beyond elevated BP, directly impacting kidney tissue and promoting fibrosis, thereby exacerbating the CKD onset and progression (Yu et al., 1998), (Borrelli et al., 2020).

This study aims to analyze the histopathological effects of high-salt intake on cardiovascular and kidney tissues and to evaluate the risk factors associated with a high-salt diet. Consequently, this study seeks to elucidate the pathological outcomes of high salt consumption on major organs, particularly the cardiovascular system and kidneys, and to confirm the detrimental effects of excessive salt intake on health.

## Methods

### Animal subjects

All procedures involving animal experimentation were approved by the Institutional Animal Care and Use Committee of the Samsung Medical Center, Sungkyunkwan University School of Medicine (approval no.: 20230817001). Male Sprague Dawley rats, aged 4 weeks, were purchased from Orient Bio Inc. and acclimatized for 1 week in the animal facility before use. To minimize handling stress, all rats were further habituated to the rat holder for more than 1 h per day for 1 week and were enrolled in the experiment at 6 weeks of age.

The control group (n = 9) received a standard diet containing 0.5% sodium chloride (NaCl), while the experimental group (n = 10) was fed a high-salt diet containing 4% NaCl (ENVIGO, TD.92034) for 12 weeks starting at 6 weeks of age. The 4% NaCl concentration was selected based on previous reports demonstrating that this dietary level is sufficient to induce vascular remodeling, renal injury, and early cardiac alterations in rodents without causing excessive systemic toxicity or mortality (Yu et al., 1998), (Wei et al., 2017), (Dahl et al., 1968). The feeding duration was set at 12 weeks to model chronic high-salt exposure, a timeframe widely used in rodent studies to allow the development of measurable histopathological and functional changes in cardiovascular and kidney tissues while minimizing confounding effects of aging (Teixeira et al., 2020), (Gomes et al., 2017).

### Experimental procedures

#### Blood Pressure (BP)

BP changes due to the high-salt diet were measured using a noninvasive tail-cuff method (CODA; High Throughput System, Kent Scientific). To reduce stress-related BP fluctuations, rats were trained in the holder 1 week before measurement. During BP measurements, rats were placed on a warming platform until body temperature stabilized at 32 °C–36 °C. Measurements were taken twice weekly, with an average of 30–45 measurements calculated per session.

#### Body weight

Each rat’s body weight was measured twice a week using a laboratory scale to monitor weight changes.

### Histological analysis

**FIGURE 1:**
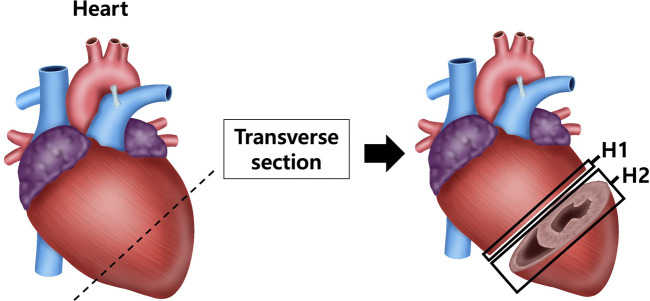
Schematic representation of heart sectioning for histological analysis. The heart was transversely sectioned at the level of the left ventricle (LV) to obtain two regions: the upper part (H1) and lower part (H2). Sections were processed for histological staining with hematoxylin and eosin (H&E) to assess ventricular wall thickness and with Masson’s trichrome (MT) to evaluate collagen fiber deposition in myocardial tissue.

**FIGURE 2:**
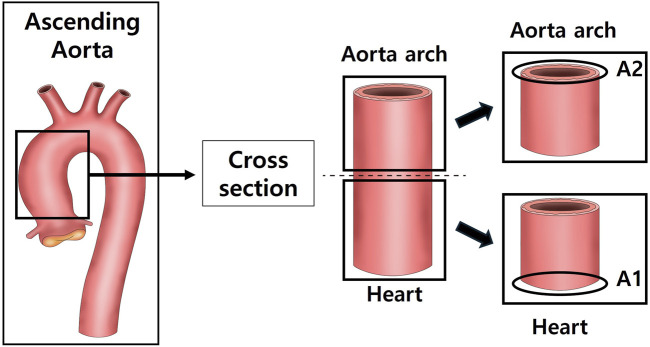
Schematic representation of aortic sectioning for histological analysis. The ascending aorta was cross-sectioned to obtain two regions: the proximal ascending aorta (A1) and the distal ascending aorta/arch transition zone (A2). Sections were stained with hematoxylin and eosin (H&E) to measure aortic wall thickness and with Masson’s trichrome (MT) to evaluate collagen deposition in the vascular wall.

**FIGURE 3:**
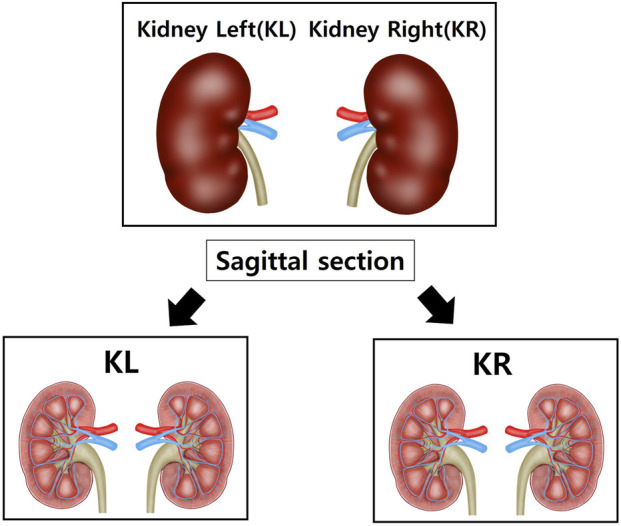
Schematic representation of kidney sectioning for histological analysis. Both kidneys were sagittally sectioned to obtain representative tissue slices. The left kidney (KL) and right kidney (KR) were processed separately to assess bilateral structural changes. Sections were prepared for histological evaluation, including hematoxylin and eosin (H&E) staining and Masson’s trichrome (MT) staining, to quantify capsular space area and collagen deposition.

After anesthetizing with isoflurane, the tissue was perfused with sterile saline, and the heart, aorta and kidneys were excised and weighed using a laboratory scale. The tissues were fixed in a 10% neutral-buffered formalin) solution. Among the fixed tissues, the heart was divided into the upper part (H1) and the lower part (H2) after a transverse section from the left ventricle (LV) (Figure 1). The aorta was cross-sectioned at the ascending aorta, dividing the proximal aorta and aortic arch side into A1 and A2 respectively (Figure 2). Kidney tissues were each sagittal-sectioned on the left and right sides, dividing the left kidney into KL, and the right kidney into KR (Figure 3).

The fixed tissues were washed for tissue staining and embedded in paraffin. The paraffin blocks were sectioned at 4 μm thickness to prepare the slides. Histological changes were observed by staining the heart, aorta, and kidneys with (hematoxylin (BBC Biochemical, 3550) and eosin (BBC Biochemical, 3610) (H&E), as well as with Masson’s trichrome stain kit (BBC Biochemical, 8400). The Scan Scope AT2 (Leica Biosystems) was used to analyze the prepared slides.

For quantitative evaluation, LV thickness was measured at 8 regions per sample, aortic wall thickness was assessed at 10 regions per sample, and renal morphology was evaluated at 20 regions per kidney sample.

### Statistical analysis

To ensure statistical rigor and account for the complexity of repeated measurements, advanced modeling approaches were employed. These methods were designed to accurately evaluate group differences while controlling for potential confounding variables. This study aimed to identify histological differences associated with a high-salt diet using both a linear mixed model (LMM) and multiple regression analyses with covariates.

In the analyses, the dependent variables included histological outcomes from the heart (left ventricle), aorta, and kidneys. -The independent variable of primary interest was dietary group (control vs. high-salt diet). Covariates included body weight (g) and age (weeks), which were incorporated to adjust for potential confounding effects. To identify which variables showed significant differences between the control and high-salt diet groups, a simple analysis of LMM was first performed, and subsequently multiple regression models were applied with body weight and age entered as covariates. Because tissue sampling was not performed at exactly the same experimental week across all individuals, statistical models were also designed to minimize variability arising from these differences in sampling time.

The choice of statistical models was based on the characteristics of the dataset. LMM was selected because it appropriately accounts for the correlation of repeated measurements within individual animals and allows random effects to capture subject-level variability. Multiple regression with covariates was additionally applied to assess group differences while controlling for confounding variables.

All statistical analyses were performed using the “lme4,” “lm,” and “margins” packages of R software.

### Multiple analysis with covariate analysis

In multivariate analysis, group differences are evaluated by adjusting for covariates, such as age (weeks) and body weight to determine significant differences between groups by controlling the variables.

#### Fixed-effects estimation

After adjusting for covariates (body weight, weeks), each independent variable (control and high-salt diet groups) was used to estimate its impact. This approach was employed to evaluate and estimate the individual effect of each variable, estimate the influence of each independent variable (control and high-salt diet groups) after adjusting for covariates (body weight, weeks), and analyze and estimate the individual influence of each variable.

#### Interaction effect

Considering the possibility that organizational changes may differ depending on the specific region (section), we additionally applied a model that included the interaction between groups and sections. In particular, the interaction effect was evaluated by section to test whether between-group differences were significant in the aorta and kidneys.

To compare the repeated measurements of each tissue in the control and high-salt diet groups, a mixed-effect analysis was performed using GraphPad Prism (Version 9.5.1). When the analysis of variance results was significant, Bonferroni’s multiple comparison test was performed as a *post hoc* test to evaluate specific between-group differences.

#### Mixed-effects analysis

This method allows the evaluation of group differences while maximizing the use of all available data, even in the presence of missing values within repeated-measures data. This approach was selected to account for the repeated-measures structure and address missing data issues required to compare the mean between-group differences.

#### Bonferroni’s multiple comparison test

This test was performed to identify which group differences were statistically significant. Adjusting the multiple comparisons. This approach minimizes errors that may occur during multiple-group comparisons and enhances reliability. The significance level for all statistical analyses was set at p < 0.05.

## Results

### Systolic blood pressure (SBP), body weight, and tissue sampling age in control and high-salt diet groups

The control group was fed a normal diet, whereas the experimental group received a high-salt diet for 12 weeks. Systolic blood pressure (SBP, mmHg) and body weight were monitored throughout the total 20-week experimental period. In addition, the age of rats at the time of group-specific tissue sampling was recorded, and the results are presented below.

The results for systolic blood pressure (SBP, mmHg) between the normal and high-salt diet groups were not statistically significant (p > 0.05, Table 1). The mean difference in body weight was slightly higher in the high-salt diet group between the two groups; however, no statistically significant changes were observed (p > 0.05, Table 1). No statistically significant changes were also observed in the age of rats between the two groups (p > 0.05, Table 1).

**TABLE 1:** Information on systolic blood pressure (SBP), body weight, and age (weeks) of rats in control and high-salt diet groups.

Parameter	Con (Mean ± SD)	High-salt (Mean ± SD)	t-value	p-value
SBP(mmHg)	122 ± 24	114 ± 19	0.78	0.44
Body Weight(g)	593 ± 72	597 ± 75	0.10	0.92
Weeks	20 ± 6	24 ± 6	1.15	0.16

Data are expressed as mean ± standard deviation (SD). Statistical analysis was performed using an independent Student’s t-test, with t-values and p-values shown. A p-value <0.05 was considered statistically significant.

### Analysis of the heart, aorta, and kidneys by group (control versus high-salt diet)

Using a linear mixed model (LMM) analysis (Table 2), pairwise comparisons were conducted between the control and high-salt diet groups for left ventricle (LV) thickness, aortic thickness and kidney capsular space. The estimated differences were 37.19 (p > 0.05) for LV thickness, 4.691 (p > 0.05) for aortic thickness, and −1.69 (p < 0.01) for capsular space. Based on these results, no significant difference in the LV and aortic thicknesses of the heart was observed between the groups, but the capsular space variables of kidney were confirmed to be significantly different between the control and high-salt diet groups and were considered as variables explaining the difference between the two groups. The observed results may have been influenced by potential confounding variables, such as age and weight, which are known to affect tissue alterations. To account for these confounders, additional multivariate analyses incorporating relevant covariates were performed. Consequently, group differences were examined more comprehensively, and interactions among variables were analyzed to enhance the accuracy and reliability of the study findings.

**TABLE 2:** Results of simple linear mixed model (LMM) analysis for heart left ventricle, aortic, and renal capsular space between control group and high-salt diet group.

Parameter	Value	Std.Error	t-value	p-value
Heart (Left Ventricle)	37.19	90.26	0.41	0.32
Aorta	4.69	4.58	1.02	0.69
Kiney (Capsular space)	−1.69	0.52	−3.23	<0.01

Dependent variables included left ventricle thickness, aortic wall thickness, and kidney capsular space area. Fixed effects were estimated for dietary group, and results are reported as regression coefficients (Value) with standard errors (Std. Error), t-values, and p-values. A p-value <0.05 was considered statistically significant.

### Histopathological alterations in the left ventricle (LV) induced by high-salt intake

To evaluate the individual effects of each variable between the two groups, a fixed-effects model was estimated with adjustments for relevant covariates, including body weight and age. The analysis revealed no statistically significant difference in left ventricle (LV) size between the high-salt diet group and the control group (p > 0.05; Table 3). In contrast, body weight exhibited a statistically significant effect on LV size (p < 0.05; Table 3).

**TABLE 3:** Fixed effects estimates for heart left ventricle thickness, aortic wall thickness, and renal capsular. space.

Parameter	Value	Std.Error	t-value	p-value
Heart (LV)				
Group (High-salt vs. Control)	33.53	80.21	0.42	0.68
Weeks	−0.55	7.57	−0.07	0.94
Body weight	1.62	0.59	2.744	<0.05
Aorta				
Group (High-salt vs. Control)	3.40	4.66	0.73	0.48
Weeks	0.05	0.03	1.33	0.20
Body weight	0.25	0.44	0.57	0.58
Kidney (Capsular space)				
Group (High-salt vs. Control)	−2.10	0.45	−4.65	<0.0001
Weeks	0.10	0.04	2.26	<0.05
Body weight	0.003	0.003	1.02	0.33

The analysis was performed using a linear mixed model (LMM) with dietary group (control vs. high-salt diet) as the main independent variable, adjusting for covariates such as body weight and age. Reported values include regression coefficients (value), standard errors (Std. Error), t-values, and p-values. A p-value <0.05 was considered statistically significant.

When examining differences based on the heart (LV) section, no significant group interaction effects was found (p > 0.05, Table 4). However, body weight remained a significant factor affecting LV size (p < 0.05; Table 4).

**TABLE 4:** Interaction effects on heart left ventricle thickness, aortic wall thickness, and renal capsular space.

Parameter	F-value	p-value
Heart (LV)		
Group (High-salt vs. Control)	0.071	0.79
Heart section	0.14	0.70
Weeks	0.01	0.95
Body weight	7.47	<0.05
High-Salt:Heart section	0.021	0.88
Aorta		
Group (High-salt vs. Control)	0.038	0.85
Aorta section	11.44	<0.0001
Weeks	1.77	0.20
Body weight	0.32	0.58
High-Salt:Aorta section	8.69	<0.0001
Kidney (Capsular space)		
Group (High-salt vs. Control)	33.25	<0.0001
Capsular space section	5.77	<0.001
Weeks	5.08	<0.05
Body weight	1.05	0.32
High-Salt:Capsular space section	4.62	<0.01

The linear mixed model (LMM) included an interaction term between dietary group (control vs. high-salt diet) and tissue section (heart regions H1/H2, aortic regions A1/A2, kidney regions KL/KR). Reported values include F-values and p-values for each interaction term. A p-value <0.05 was considered statistically significant.

Both the fixed-effects and the analysis of interaction effects in LV regions consistently indicated that body weight is a significant contributing factor to increased LV size.

**FIGURE 4:**
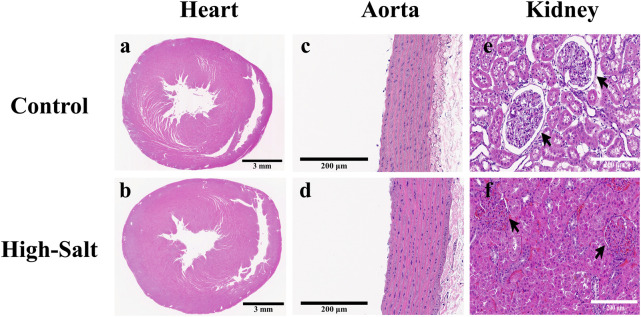
Representative H&E-stained sections of heart, aorta, and kidney tissues from control and high-salt diet (HS) groups. **(a,b)** Cross-sections of the heart showing overall myocardial structure in the control **(a)** and HS **(b)** groups. **(c,d)** Aortic wall morphology in the control **(c)** and HS **(d)** groups. **(e,f)** Kidney cortex showing glomeruli (arrows) and surrounding tubular structures in the control **(e)** and HS **(f)** groups. Note the narrowing of the capsular space and structural alterations in the HS group. Scale bars: 3 mm **(a,b)**; 200 μm **(c–f)**.

**FIGURE 5:**
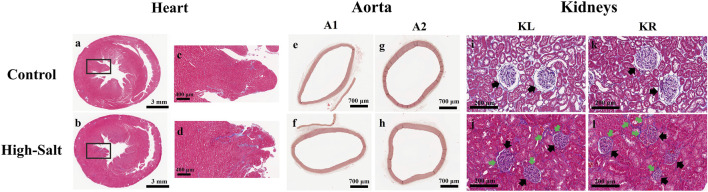
Representative histological images of heart, aorta, and kidney tissues from control and high-salt diet groups stained with Masson’s trichrome (MT). **(a,b)** Transverse sections of the heart showing myocardial collagen deposition in the control **(a)** and HS **(b)** groups. **(c,d)** Enlarged views of the boxed regions in **(a,b)**, respectively, highlighting myocardial fibers. **(e,f)** Cross-sections of the ascending aorta (A1) from control **(e)** and high-salt **(f)** groups. **(g,h)** Cross-sections of the arch ascending aorta (A2) from control **(g)** and high-salt **(h)** groups. **(i–l)** Kidney cortex sections from the control **(i,k)** and HS **(j,l)** groups. Black arrows indicate glomeruli, and green arrows highlight areas of interstitial collagen deposition. Increased collagen accumulation and tubular alterations are evident in the HS group. Scale bars: 3 mm **(a,b)**; 400 μm **(c,d)**; 700 μm **(e–h)**; 200 μm **(i–l)**.

LV thickness was assessed using heart sections stained with H&E (Figures 4a,b). In addition, collagen fiber deposition in the LV was evaluated by Masson’s trichrome (MT) staining (Figures 5a–d). Quantitative image analysis revealed no statistically significant difference in the percentage area of collagen fibers between the control and high-salt diet groups (p > 0.05). These findings indicate that high-salt intake did not induce overt myocardial fibrosis under the present experimental conditions.

**FIGURE 6:**
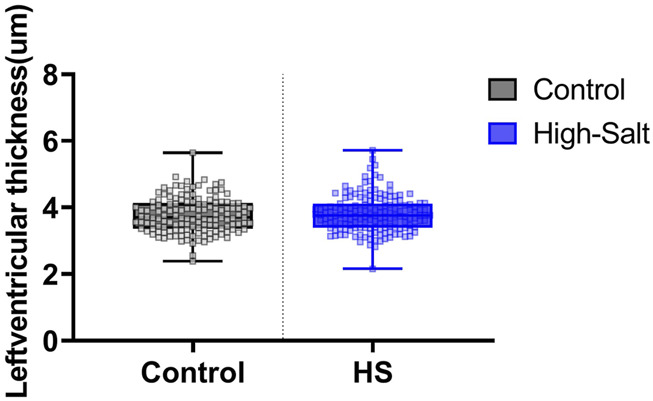
Left ventricle thickness was measured in heart tissue sections stained with hematoxylin and eosin (H&E). Quantitative analysis was performed using the Aperio ScanScope system. For each sample, measurements were obtained from eight distinct regions of the left ventricle, and the average value was used for analysis. Data are presented as violin plots with median, interquartile range, and minimum-maximum values. No statistically significant differences were observed between the control (n = 9) and high-salt diet (HS; n = 10) groups (p > 0.05).

**FIGURE 7:**
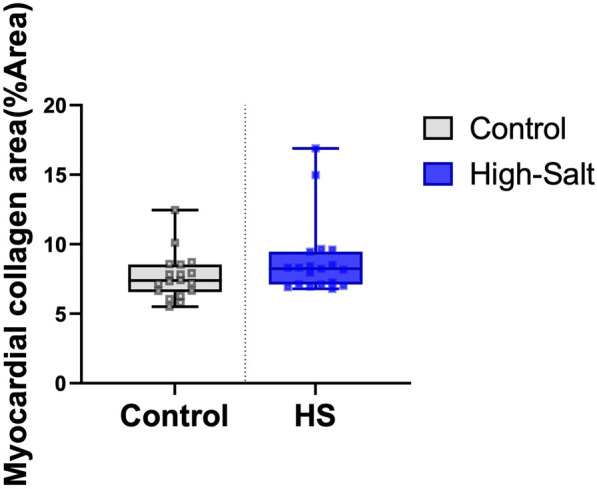
Myocardial collagen fiber content was evaluated using Masson’s trichrome (MT) staining. The percentage area of collagen fibers (%Area) was quantified in heart tissue sections from the control (n = 9) and high-salt diet (HS; n = 10) groups using ImageJ software. Data are presented as box-and-whisker plots showing median, interquartile range, and minimum-maximum values. No statistically significant differences were observed between the groups (p > 0.05).

Data were obtained from the control (n = 9) and high-salt diet groups (n = 10), with results presented as means ± standard deviation (SD; Figure 6(H&E), Figure 7(MT)).

### Histopathological alterations in aorta tissue induced by high-salt

The significance test based on fixed-effects estimation revealed no statistically significant difference in aortic vessel size between the high-salt diet group and the control group (p > 0.05; Table 3). Although no individual variable showed a significant difference between the groups, an interaction analysis was conducted to account for potential variations in aortic thickness depending on the measurement location. This analysis revealed a statistically significant interaction between the aortic section and the experimental group (p < 0.0001; Table 4).

Based on the significant results in the aortic sections, between-group comparisons at each section indicated a statistically significant difference in the A2 section (the distal part of the ascending aorta in Zone 0, which connects to the aortic arch) between the control and high-salt diet groups (p < 0.05, Table 5). In contrast, neither body weight nor experimental duration (weeks) had a statistically significant effect on aortic thickness. These findings suggest that the direction and magnitude of group differences varied by sectional location, potentially offsetting one another and thereby contributing to the absence of an overall statistically significant difference. Notably, this observation is in line with previous reports that high-salt intake can induce localized vascular remodeling and endothelial dysfunction independent of systemic blood pressure elevation (Walker et al., 2019).

**TABLE 5:** Sectional Comparison of Aortic wall thickness Between Control and High-Salt Diet Groups.

Section	Estimate (μm)	Std.Error	p-value
A1	−0.97	5.22	0.86
A2	13.71	5.22	<0.05

Estimates represent the mean difference in thickness (µm) between groups for each aortic section. A1 indicates the proximal ascending aorta, while A2 corresponds to the distal ascending aorta at the transition into the aortic arch. Values are presented as estimated differences with standard errors (Std. Error) and p-values. A p-value <0.05 was considered statistically significant.

Aortic thickness was measured using aorta sections stained with H&E (Figures 4C,D). In addition, collagen fiber deposition within the aortic wall was assessed by Masson’s trichrome (MT) staining (Figures 5e–h). Quantitative analysis revealed no statistically significant differences in the percentage area of collagen fibers between the control and high-salt diet groups in either the A1 or A2 sections (p > 0.05). These results indicate that, under the present experimental conditions, high-salt intake induced localized changes in aortic wall thickness without a corresponding increase in collagen fiber accumulation.

**FIGURE 8:**
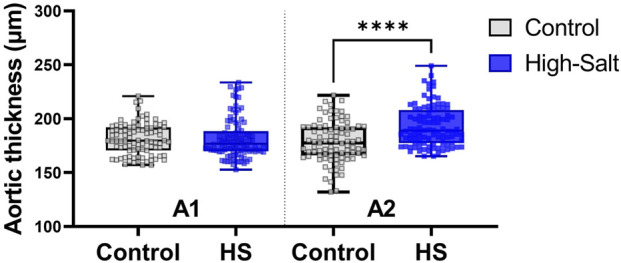
Aortic wall thickness was measured in tissue sections stained with hematoxylin and eosin (H&E). Quantitative analysis was performed using the Aperio ScanScope system. For each sample, measurements were obtained from ten distinct regions of the aortic wall, and the average value was used for analysis. Data are presented as violin plots with median, interquartile range, and minimum-maximum values. A significant increase in aortic thickness was observed in the A2 region (distal ascending aorta/transition to the aortic arch) of the high-salt diet (HS) group compared with the control group (****p < 0.0001), whereas no significant differences were detected in the Al region.

**FIGURE 9:**
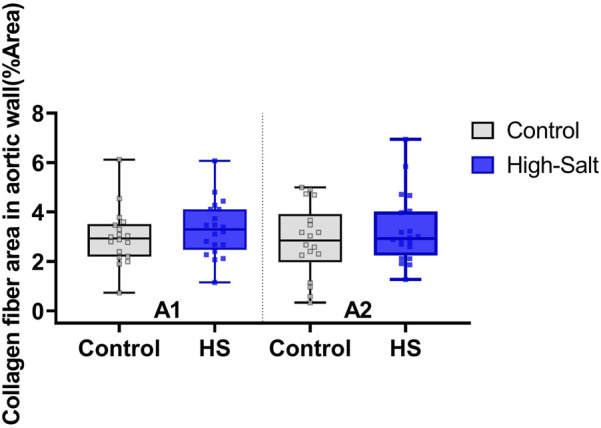
Collagen fiber content in the aortic wall was evaluated using Masson’s trichrome (MT) staining. The percentage area of collagen fibers (%Area) was quantified in two anatomical regions of the aorta: A1 (proximal ascending aorta) and A2 (distal ascending aorta/transition to the aortic arch). Data were obtained from the control (n = 9) and high-salt diet (HS; n = 10) groups using ImageJ software. Results are presented as box-and-whisker plots showing median, interquartile range, and minimum–maximum values. No statistically significant differences were observed between groups in either region (p > 0.05).

Data were obtained from the control (n = 9) and high-salt diet groups (n = 10), with results presented as means ± SD (Figure 8(H&E), Figure 9MT).

### Histopathological alterations in kidney tissue induced by high-salt

After adjusting for covariates and estimating fixed effects, the kidney capsular space in the high-salt diet group was found to be 2.10 μm smaller than that in the control group (p < 0.0001; Table 3). This finding confirms that the capsular space in the high-salt diet group was consistently narrower, with the difference being statistically significant. These results suggest that high-salt diet intake induces morphological alterations in the kidney capsular space.

Furthermore, a significant interaction effect was found when assessing group differences among different kidney sections (p < 0.01, Table 4). This result indicates that the degree of differences between the groups varied based on the level of kidneys sections. In both the left and right kidneys regions, the capsular space in the high-salt diet group was consistently estimated to be smaller than in the control group, with all differences being statistically significant (Table 6).

**TABLE 6:** Sectional comparison of renal capsular space between control and high-salt diet groups.

Section	Estimate (μm)	Std.error	p-value
RL	−2.26	0.50	<0.01
RR	−1.94	0.50	<0.01

Estimates represent the mean difference in capsular space (%Area) between groups for each kidney section. KL, indicates the left kidney, while KR, corresponds to the right kidney. Values are presented as estimated differences with standard errors (Std. Error) and p-values. A p-value <0.05 was considered statistically significant.

Kidney sections were stained with H&E, and the capsular space was subsequently measured (Figures 4e,f). For quantification, the area of the capsular space was defined as the region between the glomerular tuft and Bowman’s capsule, and its relative proportion was calculated using ImageJ software according to the following formula:

$$\%\text{Area}=\left(\text{Capsular space}/\text{Total Bowman}\mathrm{’s}\text{ capsule}\right)\times 100$$

In addition, collagen fiber deposition in kidney tissue was assessed by Masson’s trichrome (MT) staining (Figures 5i–l). Histological comparison revealed denser collagen accumulation in the interstitial regions of the high-salt group compared with the control group. Moreover, MT-stained sections demonstrated glomerular hypertrophy and tubular dilatation in the high-salt group, indicating progressive structural remodeling. Quantitative image analysis confirmed a significant increase in the percentage area of collagen fibers in both the left and right kidneys of the high-salt diet group (p < 0.0001).

These results indicate that high-salt intake not only narrows the capsular space but also promotes glomerular enlargement, tubular expansion, and excessive extracellular matrix accumulation, thereby contributing to renal fibrosis. These morphological changes are consistent with earlier findings in normotensive models indicating that high salt diet induces tubular injury, interstitial fibrosis, and a pro-fibrotic renal response independent of hypertension (Teixeira et al., 2020). Furthermore, similar glomerular hypertrophy and renal fibrosis have been reported under high-salt conditions, accompanied by upregulation of profibrotic mediators such as TGF-β1 (Yu et al., 1998).

**FIGURE 10:**
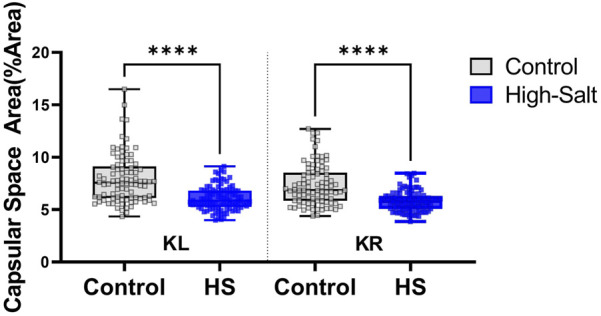
Capsular space area was measured in kidney sections stained with hematoxylin and eosin (H&E). ImageJ software was used to quantify the relative proportion of the capsular space, defined as the area between the glomerular tuft and Bowman’s capsule. The percentage area was calculated using the formula: %Area = (Capsular space/Total Bowman’s capsule) × 100. Measurements were obtained from twenty distinct regions per sample in both the left kidney (KL) and right kidney (KR). Data were collected from the control (n = 9) and high-salt diet (HS; n = 10) groups and are presented as violin plots with median, interquartile range, and minimum–maximum values. A significant reduction in capsular space area was observed in both kidneys of the high-salt group compared with controls (****p < 0.0001).

**FIGURE 11:**
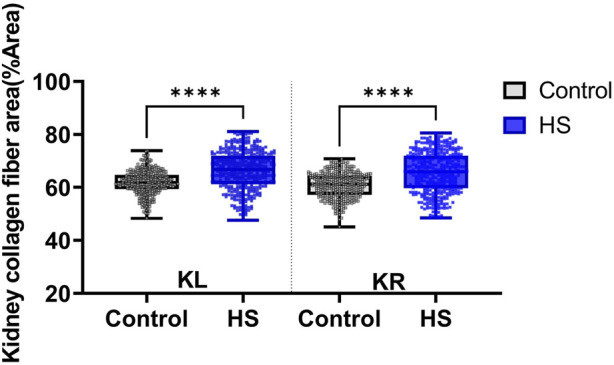
Collagen fiber deposition in kidney tissue was assessed using Masson’s trichrome (MT) staining. The percentage area of collagen fibers (%Area) was quantified separately in the left kidney (KL) and right kidney (KR) sections. Data were obtained from the control (n = 9) and high-salt diet (HS; n = 10) groups using ImageJ software. Results are presented as box-and-whisker plots showing median, interquartile range, and minimum-maximum values. A significant increase in collagen fiber area was observed in both kidneys of the high-salt group compared with controls (****p < 0.0001).

Data were collected from the control group (n = 9) and high-salt diet group (n = 10), with results expressed as means ± SD (Figure 10(H&E), Figure 11(MT)).

In conclusion, this study demonstrates that high salt intake induces localized histopathological changes in specific regions of the aorta (A2) and kidney (capsular space). In the kidney tissue, pronounced glomerular hypertrophy, narrowing of the capsular space, and tubular dilatation were observed, highlighting the detrimental effects of excessive salt consumption on renal structure. Consistent with these morphological alterations, Masson’s trichrome (MT) staining revealed a significant increase in interstitial collagen fiber deposition in both kidneys of the high-salt diet group, indicating progressive extracellular matrix accumulation and fibrosis. In contrast, no significant increase in collagen deposition was detected in the left ventricle or in the aortic wall (A1 and A2 sections), suggesting that fibrosis was not yet established in these tissues under the present experimental conditions.

These findings are consistent with previous studies that have reported renal injury and fibrosis associated with high-salt diets (Teixeira et al., 2020), (Gu et al., 2008), (Dahl et al., 1968). However, unlike earlier research indicating that high salt intake promotes LV hypertrophy (Teixeira et al., 2020), (Yu et al., 1998), (Gomes et al., 2017), no significant pathological changes in the cardiac tissue were observed aside from those associated with increased body weight.

## Discussion

A key insight from this study is the identification of early, region-specific histopathological changes in the aorta and kidney that occurred independently of significant systemic blood pressure elevation. This suggests that structural remodeling of these organs may represent an early pathogenic event triggered directly by excessive salt intake, rather than a secondary consequence of hypertension. Such an interpretation aligns with recent mechanistic studies demonstrating that high salt exposure can activate local tissue responses, including oxidative stress, endothelial dysfunction, and dysregulation of sodium transporters, which collectively contribute to vascular and renal remodeling even in normotensive conditions (Krajina et al., 2022), (Bkaily et al., 2021), (Patik et al., 2021).

In particular, the finding of capsular space narrowing accompanied by glomerular hypertrophy and interstitial fibrosis in the kidneys highlights a potential pathway by which salt intake accelerates the onset of chronic kidney disease. These morphological changes are consistent with studies reporting the activation of profibrotic mediators such as TGF-β1 (Oberleithner, 2012) and the induction of endothelial-to-mesenchymal transition (EndMT) in response to high-salt diets (Chen et al., 2023). Importantly, our results provide histological confirmation that these pathogenic pathways manifest in structural alterations detectable at relatively early stages of salt exposure.

Compared to earlier reports that focused primarily on blood pressure elevation or left ventricle remodeling, the novelty of our study lies in demonstrating that high salt intake can produce measurable remodeling in discrete vascular (aortic Zone 0) and renal (capsular space) regions without parallel cardiac hypertrophy or systemic hypertension. This distinction emphasizes the heterogeneity of salt-induced pathology across different organ systems. By integrating localized histological assessments with advanced statistical modeling, this study expands upon previous work and underscores the importance of tissue-specific evaluation in dietary salt research.

Our study has limitations in the following aspects. First, the duration of the high-salt diet exposure and the timing of tissue sampling were not standardized across individuals, making it difficult to accurately assess temporal changes. This may limit the comparison of the intensity and rate of physiological changes induced by high salt intake. Second, variations in high-salt diet concentrations were not explored, making it challenging to determine the correlation between salt intake levels and tissue alterations. Third, mechanistic investigations were not performed, including analyses of protein expression or immune cell infiltration, which could have provided deeper insights into the molecular and cellular pathways underlying vascular and renal remodeling (Wei et al., 2017), (Chen et al., 2023), (Kim and Dryer, 2021), (Vinaiphat et al., 2023). Fourth, no significant increase in systolic blood pressure (SBP) was observed between the groups, which may reflect limitations in the experimental design, sample size, or duration of salt exposure. These limitations may affect the generalizability of the findings, indicating that future studies should aim to control these variables more systematically. Future investigations should therefore integrate both structural and mechanistic approaches to better elucidate the pathophysiological processes associated with high salt intake.

In conclusion, this study demonstrated that high-salt intake induces early, localized histological alterations in the aorta and kidneys, even in the absence of significant blood pressure elevation. These findings suggest that vascular and renal remodeling may precede measurable hemodynamic changes and provide a foundation for translational strategies to mitigate salt-induced aorta and renal injury.

Future studies should stratify high-salt diet exposure into multiple durations (e.g., 4, 8, 12, 24, and 48 weeks) and systematically compare histological changes in the aorta, kidney capsular space, and glomeruli according to diet duration and animal age. Such approaches will clarify dose- and time-dependent effects and determine the reversibility of these alterations, thereby enhancing the clinical relevance of preclinical findings.

## Data Availability

The original contributions presented in the study are included in the article/supplementary material, further inquiries can be directed to the corresponding authors.
